# A clinical study of canine collagen type III glomerulopathy

**DOI:** 10.1186/1746-6148-9-218

**Published:** 2013-10-24

**Authors:** Runa Rørtveit, Anna Vigdís Eggertsdóttir, Ragnar Thomassen, Frode Lingaas, Johan Høgset Jansen

**Affiliations:** 1 Department of Basic Sciences and Aquatic Medicine, Norwegian School of Veterinary Science, Oslo, Norway; 2 Department of Companion Animal Clinical Sciences, Norwegian School of Veterinary Science, Oslo, Norway; 3 Department of Production Animal Clinical Sciences, Norwegian School of Veterinary Science, Oslo, Norway

**Keywords:** Animal model, Canine, Collagen type III, Collagenofibrotic, Dog, Glomerulopathy, Hereditary, Juvenile, Nephropathy

## Abstract

**Background:**

Collagen type III glomerulopathy (Col3GP), also known as collagenofibrotic glomerulonephropathy, is a rare renal disease with unknown pathogenesis that occurs in animals and humans. We recently described a naturally occurring canine autosomal recessive model of Col3GP, and the aim of the present work was to study the clinical features of canine Col3GP and compare with the human phenotype. In humans two different clinical syndromes with different age at onset (child- or adulthood) have been observed. In children a more aggressive course with familial occurrence is described, characterized by progressively increasing proteinuria, nephrotic syndrome, hypertension and chronic renal failure. A markedly increased serum level of the aminoterminal propeptide of type III procollagen (PIIINP) is considered a useful marker for the disease. Since Col3GP and concurrent hypocomplementemia have been observed in humans, we also aimed to investigate if hypocomplementemia was present in Col3GP affected dogs. A litter consisting of seven puppies, four Col3GP affected and three healthy unaffected, was observed from the day of birth until the affected puppies developed a mild or moderate renal azotemia.

**Results:**

During the period of observation growth retardation, increasing blood pressure, progressive proteinuria, azotemia, hypoalbuminemia, hypercholesterolemia and increased serum PIIINP were observed in all the affected dogs. Hypocomplementemia was not detected. Affected dogs were euthanized between 109 and 144 days of age, and pathological examinations revealed ascites and massive glomerular accumulations of collagen type III, consistent with Col3GP.

**Conclusions:**

Dogs with Col3GP develop juvenile chronic renal failure, preceded by nephrotic syndrome, elevated serum PIIINP and hypertension, thus have similar clinical features as the juvenile Col3GP in humans. Further studies of this naturally occurring canine phenotype may provide more information on the pathogenesis and genetics of Col3GP in both animals and humans.

## Background

Collagen type III glomerulopathy (Col3GP), also known as collagenofibrotic glomerulonephropathy, is a rare renal disease that occurs in both humans and animals. It is characterized by massive glomerular accumulations of collagen type III. In humans, two different manifestations of the syndrome have been observed, with different age at onset [1,2]. The adulthood version consists of persisting proteinuria with or without hypertension, and a slowly progressing renal impairment. The occurrence is sporadic, although it has been observed in siblings [3]. In children, a more aggressive familial phenotype is described, with progressively increasing proteinuria, nephrotic syndrome, hypertension and chronic renal failure [4]. A marked elevation of the aminoterminal propeptide of type III procollagen (PIIINP) in serum is characteristic for Col3GP in humans. PIIINP is enzymatically cleaved off during the synthesis of collagen type III and released into the blood, thus increased serum levels of PIIINP might indicate an increased metabolism of collagen type III.

Col3GP has also been reported to occur in pigs [5], dogs [6-9], monkeys [10,11] and a cat [12], with clinical signs like proteinuria and hypoproteinemia, and a variable degree of renal failure. To the authors’ knowledge blood pressure and serum PIIINP levels in Col3GP affected animals have not been reported.

Two human cases of Col3GP and concurrent factor H deficiency have been reported, and a possible connection between Col3GP and defects in the complement system has been proposed [1,13]. Deficiency of factor H leads to uncontrolled alternative pathway complement activation causing hypocomplementemia (low serum C3). In animals complement factor H deficiency has previously been described to occur spontaneously only in the porcine species; in Norwegian Yorkshire pigs [14] developing membranoproliferative glomerulonephritis type II [15].

We recently described a naturally occurring canine autosomal recessive model of Col3GP [6], documenting that the morphology of Col3GP in dogs and humans is similar. The aim of the present study was to describe the clinical features of canine Col3GP including determining serum levels of PIIINP, and compare with the clinical features of the human variant of the disease. We also aimed to investigate if hypocomplementemia is present in affected dogs.

## Methods

### Animals

A litter consisting of seven mixed-breed puppies was studied. The litter was produced by cross mating two dogs that previously had produced Col3GP affected offspring. Four of the puppies, two males and two females, developed the disease. The remaining three puppies, two males and a female, showed no clinical signs of disease during the observation period. The litter was observed from the day of birth until the affected puppies developed mild or moderate renal azotemia. When clinical signs of discomfort appeared the affected puppies were euthanized, and the healthy ones adopted into private homes at the age of ten weeks. Age at euthanasia ranged from 109-144 days. During the observation period the puppies lived in the kennel of the Norwegian School of Veterinary Science (NVH), and they were weaned at eight weeks of age. The feeding regime was standardized for all the puppies during the observation period. Commercial dry dog food for puppies was gradually introduced from three weeks of age and water was given *ad libitum*.

The study was approved by the Norwegian Animal Research Authority and performed in accordance with the National animal welfare rules and regulation for research involving animals (LOV-2009-06-19-97/FOR-1996-01-15-23).

### Clinical evaluations

The puppies were thoroughly observed every day. Every other week all the puppies went through a physical examination including measurement of blood pressure and blood analyses. Due to the small body size of the puppies, blood pressure measurements were not performed until the puppies were 25 days of age. Analyses of voided urine samples and body weight were recorded weekly.

Systemic blood pressure was measured indirectly with an oscillometric system (Cardell Veterinary Monitor 9401 BP, Sharn Veterinary Inc, Tampa, Florida), and the procedure was standardized and performed by the same trained veterinary nurse. The puppies were placed in a relaxed position, either on the floor or on the lap of the personnel. The cuff was applied below the elbow and cuff size was assessed to be approximately 40% of the circumference of the cuff site. Between three and six consecutive consistent values of systolic, diastolic and mean arterial pressure were recorded. If a measured value deviated markedly from the other values this value would be discarded and additional measurements made. The recorded blood pressure was the average of at least three successful measurements. Hypertension was diagnosed if systolic pressure exceeded 160 mmHg or diastolic pressure exceeded 100 mmHg.

Urine analysis included commercial dipstick analysis (Krub; Kruuse, Marslev, Denmark), urine specific gravity (USG) measured with a refractometer (URC-Ne, ATAGO, Tokyo, Japan), and urine protein:creatinine ratio (UPC) analyzed at the Central Laboratory, NVH.

Blood analyses included standard hematology and clinical chemistry and were analyzed at the Central Laboratory, NVH.

In addition to the four affected puppies from the studied litter, three other Col3GP affected individuals from the same pedigree contributed to the serum analyses of PIIINP and C3, and eight healthy dogs of similar age, including four from the same pedigree, were used as control animals. Venous blood was collected into plain tubes, allowed to clot at room temperature and after centrifugation the serum was transferred to plastic tubes and stored at −80°C until analysis. PIIINP was determined using a commercially available quantitative radioimmunoassay designed for measurement of PIIINP in human serum, UniQ PIIINP RIA (Orion Diagnostica, Espoo, Finland). This kit has previously been validated for measurement of PIIINP in canine serum [16]. Serum levels of C3 were assessed using a commercially available immunoperoxidase assay for determination of C3 in dog sera (ICL, Portland, OR). Both assays were performed according to the manufacturers’ instructions. Assuming normal distributions group means were compared using a two-sided t-test with unequal variances. Due to an age effect on serum PIIINP concentrations the statistical comparison regarding this analyte was performed between two subgroups (n = 5) with similar age, of healthy (mean age 102 days, range 73-141) and affected (mean age 120 days, range 80-144).

### Pathological examination

The affected puppies were autopsied immediately after euthanasia. Renal tissue samples were prepared for transmission electron microscopy in addition to light microscopy. For light microscopy, tissues were fixed in 10% buffered formaldehyde and embedded in paraffin, and 2-4 μm thick sections of renal tissue were stained with hematoxylin and eosin, periodic acid-Schiff and van Gieson, and studied in a Nikon Eclipse 50i microscope. Photomicrographs were captured with a Nikon DS-Fi1 camera using NIS Elements Basic Research software. For transmission electron microscopy, tissues were prefixed in 3% glutaraldehyde in 0.1 M cacodylatebuffer, post-fixed in osmium, dehydrated in ethanol and embedded in epon. Semithin sections were stained with toluidine blue and examined under the light microscope for selection of glomeruli. Ultrathin sections were stained with uranyl acetate and lead citrate, and the specimens were observed in a Phillips CM 10 transmisson electron microscope.

## Results

### Clinical findings

Physical examinations, body weight, blood pressure and analysis of blood and urine samples were initially without remarks for all the puppies and remained unremarkable for the healthy ones.

**Figure 1:**
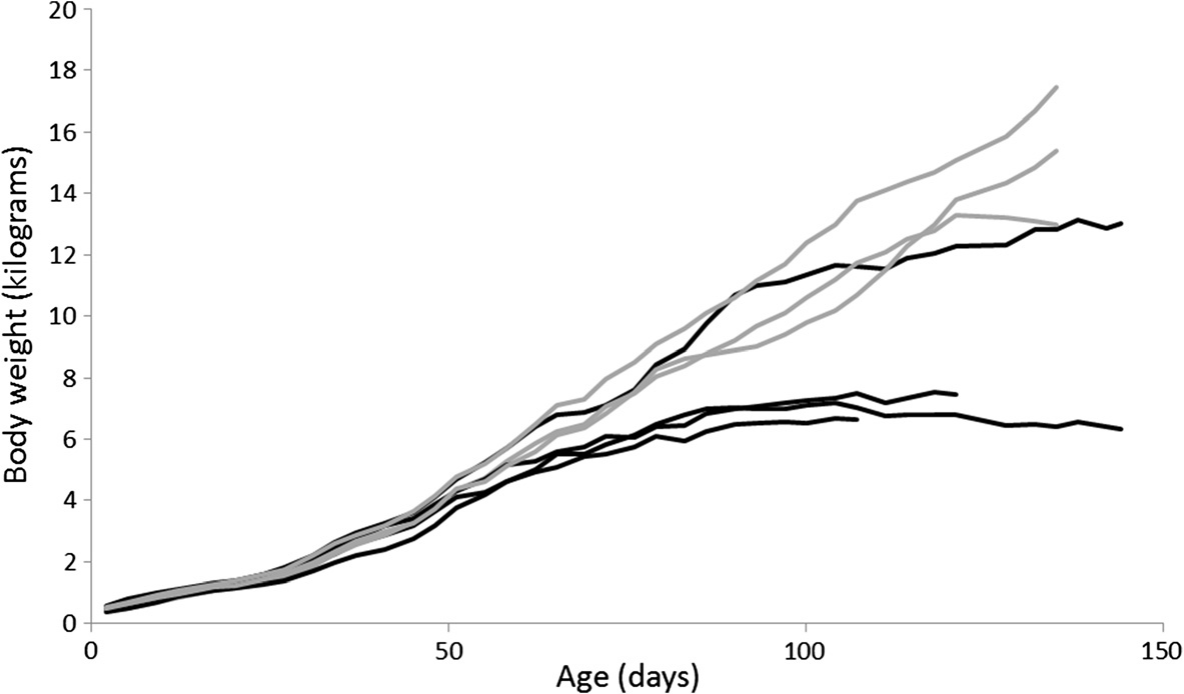
**Body weights of healthy and affected puppies during the study period.** The affected showed a decreased growth rate from around six to eight weeks of age and weight increase ceased from around 12 weeks. Black line: Col3GP affected. Grey line: Col3GP unaffected.

**Figure 2:**
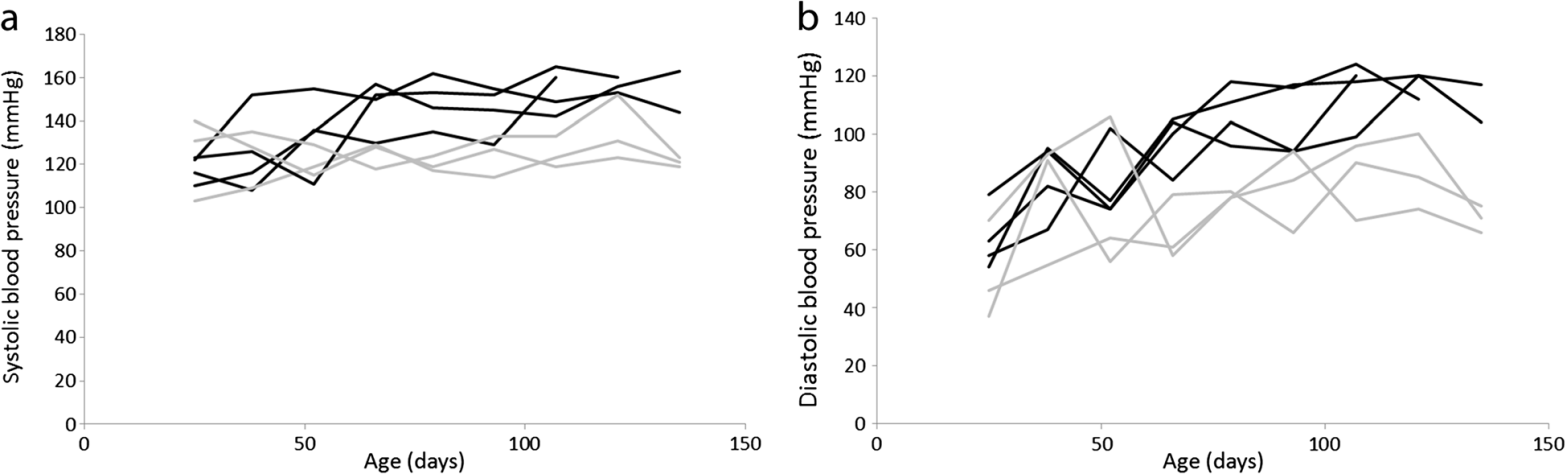
**Blood pressure in healthy and affected puppies.** The systolic **(a)** and diastolic **(b)** blood pressures in the affected puppies gradually increased during the study period and stabilized at a higher level than the healthy siblings. Black line: Col3GP affected. Grey line: Col3GP unaffected.

**Figure 3:**
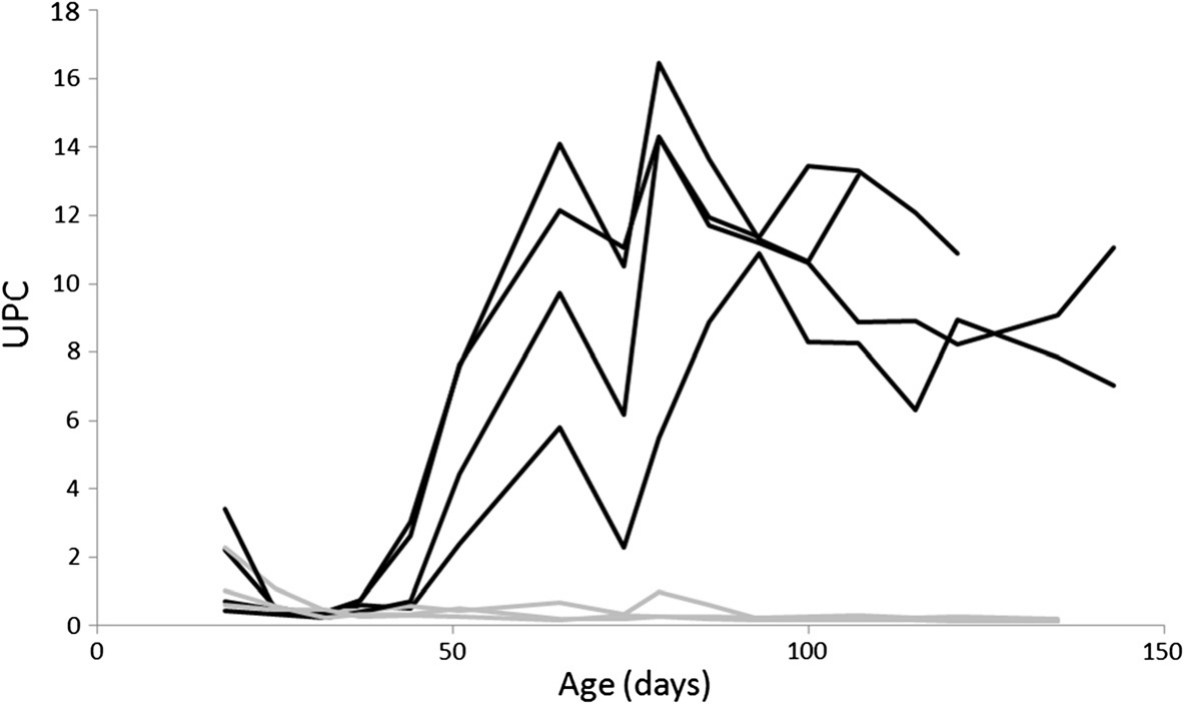
**Urine protein:creatinine (UPC) ratio in affected and unaffected puppies during the observation period.** From around 50 days of age UPC increased rapidly in affected puppies, and the proteinuria was persistent at a high-magnitude level. Black line: Col3GP affected. Grey line: Col3GP unaffected.

From six to eight weeks of age the affected dogs showed a decreased growth rate and became thinner than their healthy siblings, and from 12 weeks of age weight increase ceased (Figure 1). Increases in systolic and diastolic blood pressures were observed (Figure 2a and b), and higher blood pressure values were found in affected dogs compared to the healthy siblings (Table 1). Proteinuria was first detected by analysis of UPC on day 44 (two dogs) and 51 (two dogs). The proteinuria was persistent and rapidly increased before stabilizing at a high level (Figure 3), and was markedly higher than in the healthy littermates (Table 1). The ability to concentrate urine gradually decreased. After approximately 90 days of age the USG values of the affected puppies did not exceed 1,030, and at the last evaluation before euthanasia USG varied from 1,018 to 1,023. Urine dipstick analysis showed, in addition to proteinuria, occasional mild hematuria and glycosuria in absence of hyperglycemia.

**Table 1:** UPC, serum albumin, SCr and BP in healthy and affected puppies at various time points during the study period

		**UPC**				**Serum albumin (g/L)**				**SCr (μmol/L)**				**Systolic BP/diastolic BP (mmHg)**			
	**Time point**^**a**^	**1**	**2**	**3**	**4**	**1**	**2**	**3**	**4**	**1**	**2**	**3**	**4**	**1**	**2**	**3**	**4**
**Affected**^b^	mean	1.7	10.4	10.9	8.5	19	18	16	17	40	51	131	174	116/64	146/96	156/115	154/111
	SD	1.4	3.1	2.7	0.9	1	5	2	1	2	5	41	42	7/11	14/11	12/11	13/9
																	
**Healthy**^c^	mean	1.3	0.4	0.2	0.1	21	29	32	34	42	51	63	72	125/51	125/66	125/88	121/71
	SD	0.9	0.3	0.1	0	1	1	2	2	1	5	3	3	19/17	6/11	7/14	2/5
																	
**P-value**^d^		0.67	0.01	0.004	0.05	0.06	0.02	<0.001	0.003	0.12	0.98	0.03	0.18	0.60/0.34	0.03/0.02	0.01/0.04	0.18/0.07
																	

^a^ Time point 1, age 17-25 days; time point 2, age 65-66 days; time point 3, age 107 days; time point 4, age 135 days.

^b^ n = 4 at time point 1-3 and n = 2 at time point 4.

^c^ n = 3 at all time points.

^d^ Group means were compared using a two-sided t-test with unequal variances.

UPC, urine protein:creatinin ratio; SCr, serum creatinine; BP, blood pressure; SD, standard deviation.

**Figure 4:**
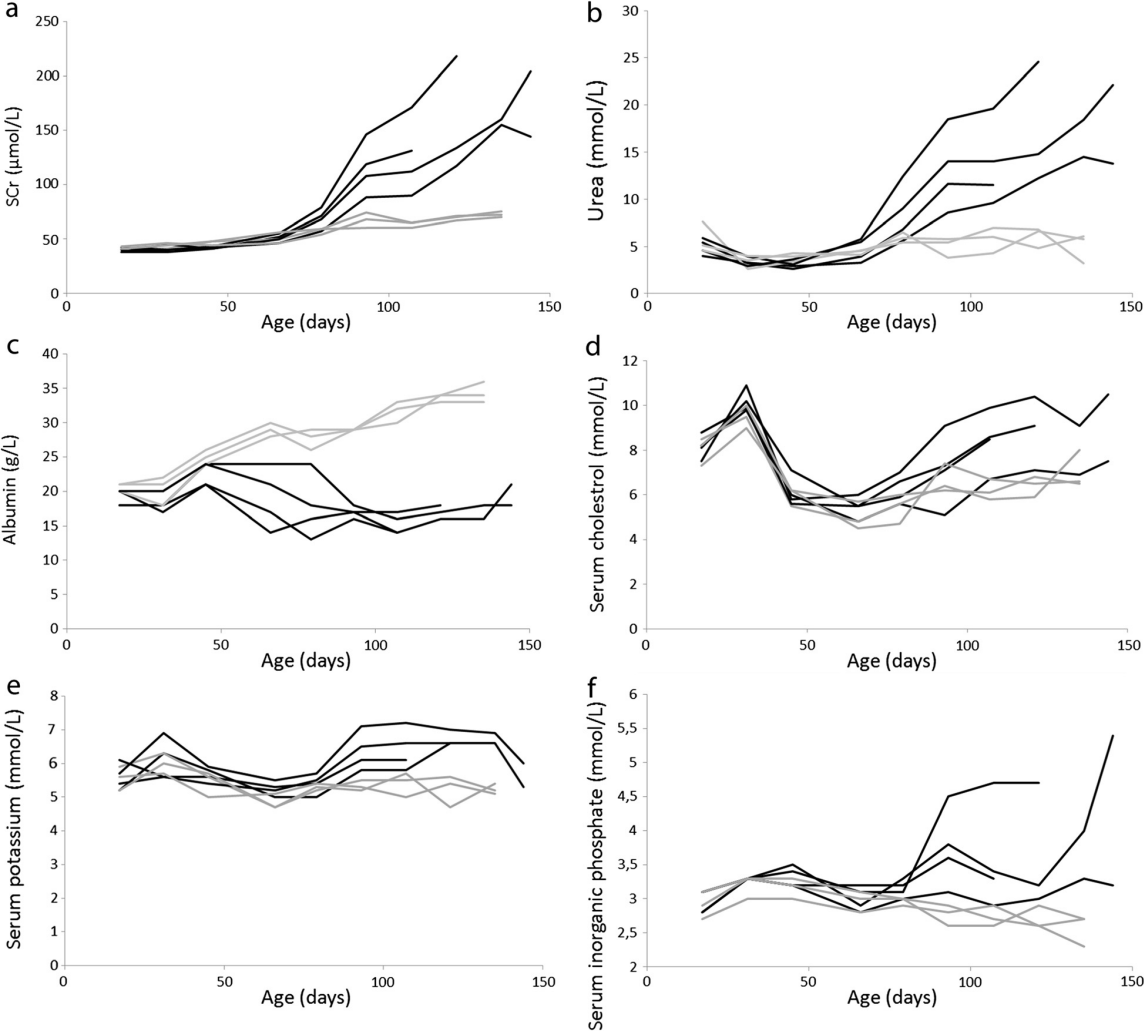
**Determination of serum clinical chemistry in affected and unaffected puppies during the observation period.** Regarding the affected puppies serum concentrations of creatinine **(a)** and urea **(b)** gradually increased from around 70 days of age. Decreased serum albumin concentrations **(c)** were detected from around 50 days of age, and increased serum cholesterol **(d)** from around day 100 (in three of four affected). Increased serum concentrations of potassium **(e)** and inorganic phosphate **(f)** were observed. Black lines: Col3GP affected. Grey lines: Col3GP unaffected.

Serum analysis revealed progressively increasing creatinine and urea values (Figure 4a and b, Table 1), decreasing albumin values (Figure 4c, Table 1), and an increased cholesterol level in three of the puppies from approximately 100 days of age (Figure 4d). Elevated serum levels of potassium (Figure 4e) and inorganic phosphate (Figure 4f) were also observed.

**Figure 5:**
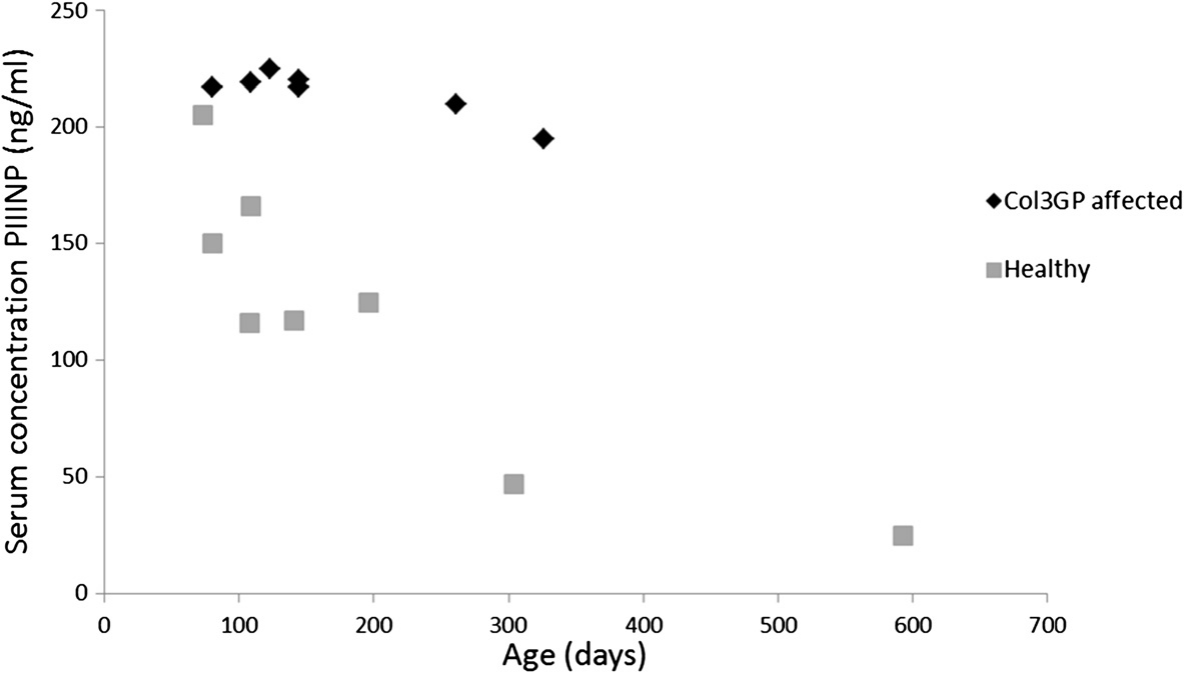
**Serum concentrations of precollagen III peptide (PIIINP) in seven affected and eight healthy controls.** The affected dogs revealed significantly higher serum levels than the healthy controls (t-test, P = 0,01). In the control group younger puppies had higher levels than the older ones.

**Figure 6:**
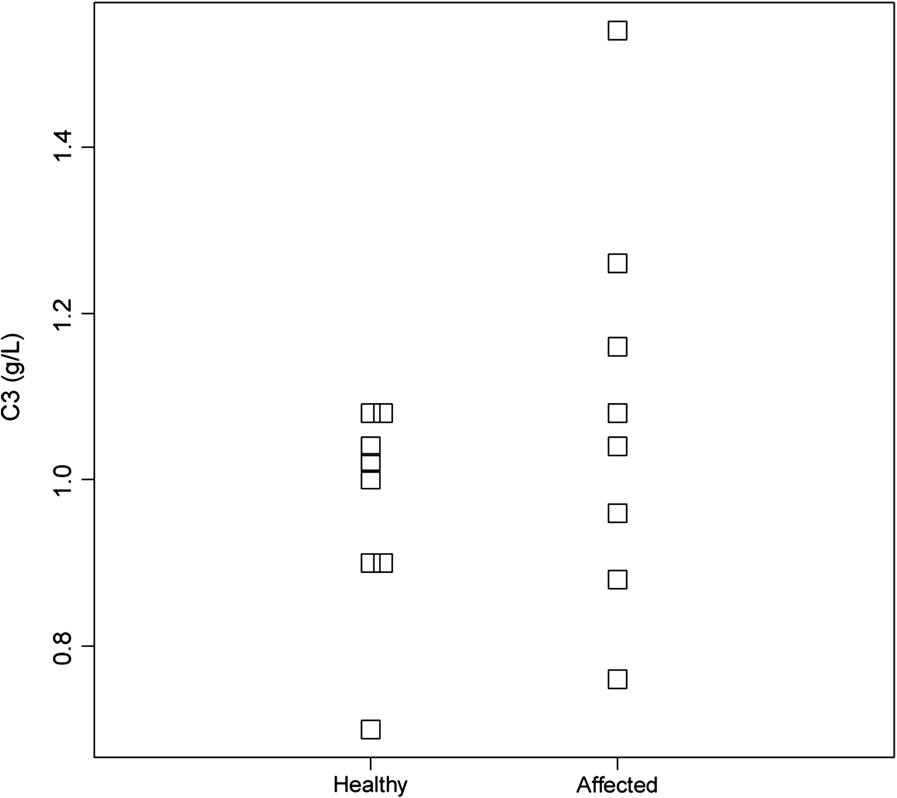
**Serum concentrations of C3 in 7 affected and 8 healthy control dogs.** There was no significant difference in serum levels of C3 between the two groups (t-test, P = 0,24).

Serum concentrations of PIIINP in seven affected and eight control animals are shown in Figure 5, and serum levels of PIIINP were significantly higher (t-test, P = 0,01) in the affected dogs (mean 220 ng/ml, standard deviation 3 ng/ml) than in the controls (mean 151 ng/ml, standard deviation 37 ng/ml). Younger puppies in the control group had higher levels of serum PIIINP than the older ones. There was no significant difference between serum levels of C3 in affected and control dogs (t-test, P = 0,24) (Figure 6).

Affected puppies were euthanized at 109 (n = 1), 123 (n = 1) and 144 (n = 2) days of age.

### Pathological findings

**Figure 7:**
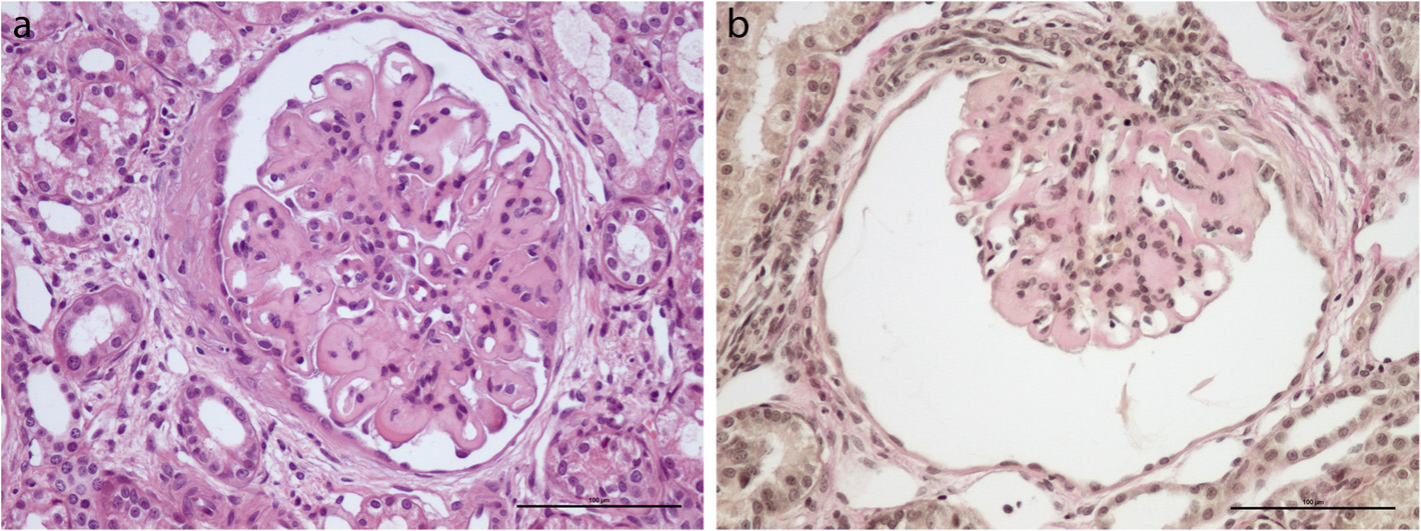
**Light microscopic appearance of affected glomeruli. (a)** The mesangium and the capillary walls are expanded by deposited extracellular eosinophilic fibrillar material (hematoxylin and eosin, ×20). **(b)** The abnormal glomerular material stain red using van Gieson stain, suggesting a collagenous nature of the deposited material (van Gieson, ×20). Bar = 100 μm.

Necropsy revealed reduced muscle mass and a moderate ascites in all four affected puppies. The kidneys were pale and firm with granular surfaces. Light microscopic studies showed diffuse glomerular lesions consisting of a severe expansion of the mesangium and the capillary walls by a homogenous eosinophilic material, and a variable degree of mesangial and endocapillary hypercellularity (Figure 7). There was mild to moderate tubular atrophy and severe periglomerular and moderate interstitial fibrosis. Occasionally interstitial infiltrations of inflammatory cells were found. By transmission electron microscopy massive glomerular accumulations of fibrillar collagen was identified in the subendothelial and mesangial space, consistent with Col3GP.

## Discussion

The Col3GP affected puppies in the present study developed nephrotic syndrome, characterized by heavy proteinuria, hypoalbuminemia, hypercholesterolemia and ascites. The proteinuria was severe and of glomerular origin [17] and the protein loss rapidly exceeded the protein synthesizing capacity, causing hypoalbuminemia. The pathological findings were consistent with Col3GP and identical to the lesions previously described in other dogs from the same pedigree [6].

Previously, three case reports describing canine Col3GP have been published [7-9]. One report describes three puppies from a litter of Newfoundland dogs, two of them two months old and the third one year old, that developed renal disease characterized by growth retardation, anorexia and proteinuria. The puppies were euthanized due to bad prognosis, and characteristic renal lesions of Col3GP were disclosed by light microscopy and transmission electron microscopy. However, immunohistochemistry to confirm the presence of collagen type III in the glomeruli was not performed. In a previous litter from the same dam and sire, two of the puppies had died at a young age, strongly supporting a hereditary disease [9]. The two other case reports describe a seven month old mongrel dog with growth retardation and depression [8], and a Shiba Inu that presented with anorexia at three years of age [7]. Both cases had a mortal outcome and at necropsy the dogs had pathological changes characteristic of Col3GP. All the described cases showed increased serum creatinine, urea, hypoalbuminemia and proteinuria. With exception of the three year old Shiba Inu, all cases showed juvenile-onset renal disease, in accordance with the litter in the present study, and the four previously reported cases from the same pedigree [6].

In humans, two kinds of Col3GP have been described, one in children and one in adults [1,2]. In children the disease tends to be more aggressive with a familial occurrence. Gubler et al. reported ten cases [4], including three pairs of siblings. The patients were from four months to 13 years of age at presentation. All showed proteinuria, six had microscopic hematuria, five were hypertensive, three were anemic and four developed renal insufficiency. In nine of the ten cases the disease followed an unremitting course [4]. The canine variant of Col3GP, with proteinuria, hypertension and early occurrence of renal impairment closely resembles the childhood version of Col3GP. However, hematuria and anemia seems to be more common in human cases than in affected dogs.

Elevated serum levels of PIIINP in Col3GP affected dogs compared to healthy age-matched controls were observed in the present study. Increased serum PIIINP is well described in Col3GP affected humans [18-22], but has so far not been reported in animals. Regarding the control group, the younger puppies had higher serum PIIINP concentrations than the older ones, and the serum levels declined with increasing age at sampling. This coincides with studies in humans [23] and dogs [16] showing that serum levels of PIIINP are higher in young growing individuals than in adults. The effect of age on serum levels of PIIINP limits its value as a marker of disease in young individuals, and emphasizes the need for age-specific reference intervals. This problem was also reflected in the present study where the differences in serum levels between affected and unaffected dogs became more apparent with increasing age. The serum levels were determined using a human kit which was validated by Schuller et al. for measurements of canine PIIINP [16]. The majority of the samples exceeded the range for which the kit was validated and therefore further validation is needed to precisely determine serum concentrations of PIIINP in dogs. However, since all the samples were treated in the same manner the results may be regarded as valid for comparison between the groups. Schuller et al. did not show differences in serum levels of PIIINP in dogs with chronic renal failure compared to healthy dogs [16]. In human cases, serum levels vary strongly. In patients with chronic renal failure a doubling was seen compared to healthy controls [24], and in patients with Col3GP a hundredfold increase has been observed [25]. The pathological significance of the markedly elevated serum PIIINP in Col3GP affected humans and dogs compared to other types of chronic renal failure is unknown. Glomerular collagen type III synthesis occurs in Col3GP exclusively, however, in chronic kidney disease, regardless of cause, the amount of collagen type III synthesized in the renal interstitium is often even more extensive. Further studies on affected dogs should be performed to clarify the pathological mechanisms of this disease, including increased serum PIIINP.

Two human cases of Col3GP with concurrent factor H deficiency resulting in hypocomplementemia have been described, suggesting an association between Col3GP and defects in the complement system [1,13]. In the present study there was no difference between levels of C3 in affected dogs and healthy controls, excluding associated hypocomplementemia.

## Conclusions

The present study describes the clinical signs of canine Col3GP, characterized by nephrotic syndrome, increased serum PIIINP and hypertension. The clinical course progressed to chronic renal failure, and resembled Col3GP occurring in children. Associated hypocomplementemia was excluded. Further studies of this naturally occurring canine phenotype may have the potential to provide more information on the pathogenesis and genetics of Col3GP in both animals and humans.

## Abbreviations

Col3GP: Collagen type III glomerulopathy; NVH: Norwegian School of Veterinary Science; PIIINP: Precollagen III propeptide; UCP: Urine protein:creatinin ratio; USG: Urine specific gravity.

## Competing interests

The authors declare that they have no competing interests.

## Authors’ contributions

RR-participated in the design of the study and the acquisition of clinical and pathological data, carried out the immunoassays and drafted the manuscript. AVE-participated in the acquisition of the clinical data and helped drafting and reviewing the manuscript. RT-participated in the acquisition of data and helped reviewing the manuscript. FL-participated in the design of the study and helped reviewing the manuscript. JHJ-conceiver of the study and participated in the design of the study, the collection of pathological data and helped reviewing the manuscript. All authors read and approved the final manuscript.

## Authors’ information

RR-DVM PhD student. AVE-DVM PhD Associate professor. RT-DVM PhD Associate Professor. FL-DVM PhD Professor. JHJ-DVM PhD Associate professor.
